# Associations between Children’s Risky Play and ECEC Outdoor Play Spaces and Materials

**DOI:** 10.3390/ijerph18073354

**Published:** 2021-03-24

**Authors:** Ellen Beate Hansen Sandseter, Ole Johan Sando, Rasmus Kleppe

**Affiliations:** 1 Department of Physical Education and Health, Queen Maud University College of Early Childhood Education, 7044 Trondheim, Norway; ojs@dmmh.no; 2 Department of Early Childhood Education, Faculty of Education and International Studies, Oslo Metropolitan University, 0167 Oslo, Norway; rask@oslomet.no

**Keywords:** risky play, free play, outdoor environments, early childhood education, play materials

## Abstract

Children spend a large amount of time each day in early childhood education and care (ECEC) institutions, and the ECEC play environments are important for children’s play opportunities. This includes children’s opportunities to engage in risky play. This study examined the relationship between the outdoor play environment and the occurrence of children’s risky play in ECEC institutions. Children (*n* = 80) were observed in two-minute sequences during periods of the day when they were free to choose what to do. The data consists of 935 randomly recorded two-minute videos, which were coded second by second for several categories of risky play as well as where and with what materials the play occurred. Results revealed that risky play (all categories in total) was positively associated with fixed equipment for functional play, nature and other fixed structures, while analysis of play materials showed that risky play was positively associated with wheeled toys. The results can support practitioners in developing their outdoor areas to provide varied and exciting play opportunities.

## 1. Introduction

Contrary to the ideals expressed in the UN Convention on the Rights of the Child [1], where children’s right to autonomy, agency, and free play are emphasized, children’s lives have become increasingly regulated and controlled [2,3,4,5,6]. Recent research indicates that, over recent decades, children are left with less opportunities for free play, especially outdoors (e.g., [6,7,8,9,10]). Children’s spend more time indoor in sedentary activities, and less time in outdoor play and vigorous physical activity [11]. Similarly, research on outdoor play indicate that children rarely get opportunities to learn and master risk on their own account [9,12,13,14,15,16]. There are robust indications that children willingly seek out risky play, and it is suggested that the feeling of exhilaration is one central motivation for this behavior [17,18]. Additionally, children may experience feelings such as enjoyment, pride, achievement, and good self-esteem when they master new challenges, adding to the potential rewards of engaging in risky play [19,20,21,22].

### 1.1. Risky Play

A common definition of risky play is: “thrilling and exciting forms of physical play that involve uncertainty and a risk of physical injury” [19]. Eight categories of risky play have been established through observations and interviews with children and ECECs [23,24,25]: (1) play with great heights—danger of injury from falling, such as all forms of climbing, jumping, hanging/dangling, or balancing from heights; (2) play with high speed—uncontrolled speed and pace that can lead to a collision with something (or someone), for instance bicycling at high speeds, sledging (winter), sliding, running (uncontrollably); (3) play with dangerous tools—that can lead to injuries, for instance axe, saw, knife, hammer, or ropes; (4) play near dangerous elements—where children can fall into or from something, such as water or a fire pit; (5) rough-and-tumble play—where children can harm each other, for instance wrestling, fighting, fencing with sticks; (6) play where children go exploring alone, for instance without supervision and where there are no fences, such as in the woods; (7) play with impact—children crashing into something repeatedly just for fun; and (8) vicarious play—children experiencing thrill by watching other children (most often older) engaging in risk. Risky play and risk-taking activities are found in a great span of ages, including 1- to 3-year-olds [25], 4- to 6-year-olds [23], and 4- to 13-year-olds [21].

The possible benefits of risky play regarding children’s development have been in several researchers’ interest in the last few decades. Some of this research indicates that risky play may increase children’s physical activity [26], improve motor/physical competence [27,28], increase spatial and perceptual skills [29], and enhance their ability to assess and manage risk appropriately [30,31,32]. Children seem to have clear strategies for reducing the potential harm in exposing themselves to risk in play [33,34,35]. Brussoni et al. [26] found mainly positive outcomes in their systematic review on risky outdoor play and children’s health. On the other hand, being restricted from the experiences and learning that risky play provides might increase the chance of anxiety, both in childhood [36,37] and in adolescence and adulthood [38,39,40,41,42].

### 1.2. Risky Play and the Play Environment

The physical play environments in ECEC institutions are important for how and what kinds of play children can engage in. Several studies have shown how physical environments influence children’s play, both regarding types of play, physical activity levels, well-being, involvement, creativity, diversity, and social interactions [12,43,44,45,46,47,48]. Studies have also investigated the relationship between children’s play preferences and the physical environment (play environment) available. These studies clearly show how features in the play environment both are preferred by children and utilized by children in their play. Children tend to prefer spaces that afford challenges, manipulation and place-making, such as equipment for climbing, spaces for ball games, scooters, rock walls, and rocks to climb on, as well as natural elements such as flowers and green plants, and loose materials such as twigs, leaves, stones, sand, dirt, various tools, and toys [49,50,51]. Research has also found that children prefer non-standardized and more challenging play environments over safer and more standardized play environments [52,53]. Nevertheless, few studies have explored how children use the physical outdoor play environment to engage in different forms of risky play. Research on risky play is a relatively new research field and is dominated by research within the ECEC context [54,55].

Some studies focus directly on the association between the physical environment and children’s risky play through observation of how children use the environment. Based on a taxonomy of affordances for risky play, Sandseter [56] studied outdoor play spaces and children’s risky play in an ordinary preschool compared to a nature and outdoor preschool in Norway. Risky play happened in equal amount in both types of preschool, but Sandseter found there was higher risk involved in children’s play in the nature and outdoor preschool. Sandseter did not study in detail where children engaged in different kinds of risky play, but the results indicate that much of play in great heights was in trees, on the roof of play-huts or in climbing towers, while play with high speed happened in swings, sledging on snow and slides or riding bicycles. Bundy et al. [12] conducted an intervention study in primary schools in Sydney, Australia, and introduced loose materials such as car and bike tires, hay-bales, cardboard boxes, plastic barrels and water containers, crates, and wooden planks to the school playground. Their results showed that children’s physical activity increased, but teachers expressed concern about the play being riskier after the intervention.

In another Australian study, Little & Eager [13] highlights the importance of meeting children’s desire for stimulating and challenging play opportunities with attractive play spaces. Their findings indicate that children, to a varying degree, utilize fixed playground equipment for risky play and that children preferred activities that involved the sensations of height and speed [13]

Kleppe [57] compared how the physical environment in two ordinary ECEC centers and a nature center provided opportunities for risky play for 1- to 3-year-olds (*n* = 39). Opportunities for risky play were assessed in each center utilizing the eight risk categories as described above. The three centers were also assessed with ITERS-R ((ITERS-R (Infant-Toddler Environment Rating Scale—Revised edition). ITERS-R is a standardized assessment tool, applied in varied cultural contexts [58,59], and a well-established measurement for ECEC-quality [60]. The study showed that, out of the three, the one center that ranked highest on the standardized measurement (one of the ordinary centers) also provided the most, and the most varied, opportunities for risky play for 1- to 3-year olds. The nature center surprisingly provided less varied opportunities for risky play than the high-ranking center, e.g., no opportunities for playing with speed during summer. The physical provision was also reflected in more observed risky play in the high-ranking center compared to the other centers. The study was conducted in Norway, and the fact that a high-ranking center provides for risky play probably reflects a cultural appreciation for this aspect of play.

Recently, Obee, Sandseter, and Harper [61] reported results from a case study of one ECEC institution in Norway, including qualitative and quantitative observations. The results showed that stable structures, moveable structures and weather features afforded various kinds of risky play. Stable structures such as climbing walls, climbing structures, ledges and swings afforded play with great heights, while hills, trails, swings, and flat surfaces afforded play with high speed. Rough-and-tumble play usually happened on flat surfaces or hills. Moveable features such as loose materials afforded play with great heights (children building and playing on high constructions), while wheeled toys, sledges, skis, and mats afforded play with high speed, and loose materials, rocks, sticks, chains and ropes afforded rough-and-tumble play. Weather features such as frost, ice and snow afforded high-speed and rough-and-tumble play. This study shows interesting results on the association between affordances in children’s play environment and the types of risky play they engage in but is limited by being a case study of only one ECEC institution and a small number of children (*n* = 28 in qualitative observations and *n* = 11 in video observations).

Even though research on children’s risky play has grown over the last decades, there is a lack of large-scale studies with quantitative data on how risky play is connected to features in the physical play environment.

### 1.3. Aim of the Study

Based on the literature showing that risky play has developmental benefits and that physical play environments are important for children’s opportunities of engaging in risky play, this study aims to examine the relationship between outdoor play spaces and play materials for the occurrence of children’s risky play. Globally, children spend an increasing amount of time in ECEC institutions [62], and the physical ECEC play environment is therefore of particular interest. As such, the research question is: In what way are ECEC outdoor play spaces and materials associated with 3- to 5-year-olds’ engagement in and types of risky play?

## 2. Materials and Methods

The present study is a sub study within the large-scale project called Competence for Developing Early Childhood Education and Care Institutions’ Indoor and Outdoor Environments (EnCompetence), funded by the Research Council of Norway. The EnCompetence project focused on how children utilize physical environments when they engage in free play, collecting data from randomized video observations at two data points (fall 2017 and fall 2018). In this project, free play referred to situations in which children themselves could decide what to do, where to be and with whom to interact.

### 2.1. Participants

Eight ECEC institutions were selected to participate in the study. The selection of institutions was done strategically, and with an aim to include different types of institutions in terms of the size, environment, location and age of the spaces. Selected institutions were located in the south (*n* = 4), middle (*n* = 3) and north (*n* = 1) of Norway, had an average of 85 children (Min = 56, Max = 117) and were built between 1989 and 2016 (Mean = 2007). The ECEC institutions’ outdoor environments ranged from small (800 square meters) urban environments with mainly asphalt and rubber surfaces to large (13.000 square meters) environments with natural elements like forest areas, hilly terrain, bushes, and natural materials. On one hand, all the playgrounds were unique and had different characteristics in terms of size, topology and inclusion of nature. On the other hand, playground features were quite similar, and all of the outdoor spaces included fixed playground equipment such as swings, slides, sandpits and climbing equipment, and play materials like tricycles, buckets, cups, and spades.

Five girls and five boys were randomly selected from each institution among the children whose parents gave informed consent for participation. Eighty (80) children participated in T1, the first data collection in 2017, while seventy-nine (79) children participated in T2, the second data collection in 2018. Because there were six dropouts (due to change of ECEC institution or ethical considerations) from T1 to T2, the six new children were randomly selected to replace the dropouts. In the present analysis, the sample consisted of 86 children, 74 children participating in both T1 and T2, and 12 children participating in either T1 or T2. In the total data material, 51% of the observations were of boys, and 49% were of girls. Children’s mean age was 3.8 years (*SD* = 0.6) at T1 and 4.7 years (*SD* = 0.6) at T2.

### 2.2. Procedure and Data

The data collection, both T1 and T2, followed a strict protocol where observations were video recorded in a similar way in all ECEC institutions. This procedure ensured a random sampling of observational sequences. On each day of data collection, two children were randomly selected for observation. During the day, each child’s free outdoor play was recorded in two-minute sequences, with a total of six sequences. Following the protocol, each child was filmed for two minutes alternately with a six-minute break to switch between the two children. Sensitive situations such as toileting or changing clothes were not video recorded, and observation would be postponed until the situation was no longer sensitive. The project researcher wrote field notes and ensured that the protocol was followed, while a co-researcher (ECEC teacher) from each institution was responsible for filming.

Six observations of 80 children at two data points would, all together, have included 960 observations. Nevertheless, because of some missing observations, the final sample included only 935 video observations. The missing observations constituted 25 observations (2.6%). In some of these situations, children were sick, picked up early, and in some the child was preoccupied with the recording equipment, the data collectors experienced a technical or human error. The final sample reflected a fairly equal distribution of observations at the two data points, with 471 observations at T1 and 464 at T2. The mean duration of the 935 video observations was 122 s (*SD* = 5).

### 2.3. Ethical Considerations

Doing research with children calls for special ethical considerations [63]. The researcher needs to receive informed consent, not only from the parents, but also from the children themselves. This is required before the research starts and also before each observation. The co-researchers already knew the children and were therefore responsible for explaining to the them, in a way they would understand [64]. Each child was observed during one day at each of the two data collections. At the start of each day the child was explained what being observed meant for them, and that they could tell or in other ways express to the co-researcher that they did not want to be observed at any time during the day. The researchers were also very conscious to avoid recording children in sensitive situations or when children expressed being uncomfortable with being observed.

The study was approved by the Data Protection Official for Research in Norway.

### 2.4. Coding of Risky Play

Risky play was coded using the Observer XT 12.5 behavior coding (Noldus), analysis and management software for observation data [65]. This software allows for second-by-second coding of videos. This means coders were able to code instances and duration of the various types of risky play. Three assessors independently coded a part of the video material according to recent categories of risky play [54]:

1. Play with great heights—e.g., where children climb trees, climbing towers, play-hut roofs. Or jump down from high places such as roofs, play equipment platforms, jumping between tables, etc.
2. Play with high speed—e.g., where children slide down slides or hills, swing at high speed, or roll down steep hills sitting on a tricycle, car toy or doll trolley, etc. Cycling, sliding, or swinging at low speed was not considered risky play.
3. Play with dangerous tools—e.g., where children play with ropes, hammers and nails, whittle with knives or use saws and axes, etc. Using kitchen knives for e.g., sandwich spread was not considered risky play.
4. Play near dangerous elements—e.g., where children play near dangerous elements such as steep cliffs, deep water, fire pits, etc.
5. Rough-and-tumble play (R&T)—e.g., where children engage in play fighting, play wrestling, play fencing, chase-and-catch play, etc.
6. Play where children go exploring alone—e.g., where children are allowed to wander off into the forest or the neighborhood without the constant supervision of staff.
7. Play with impact—e.g., where children repeatedly crash their tricycles, trolleys, or other wheeled toys into the fence or a wall, or where they crash the swing into the pole of the swing set, etc.
8. Vicarious play—e.g., where children observe other children taking risks in play, and where the observing child shows clear signs of being exhilarated by what he or she observes.

After coding the 935 observations, a total of 238 observations containing risky play was identified. Agreement on coding between assessors was checked by randomly selecting 106 observations to be reviewed by one of the other assessors among the 238 observations with risky play (45%). In 76 of these observations (72%), no comments on the initial coding were made. In 22 of the observations (21%) one of the assessors made a comment on the point of starting or stopping the coding of a specific category, and in eight observations (7%) one of the assessors questioned if the chosen category of risky play was most the appropriate. The 30 observations with comments were reviewed jointly by all three assessors to discuss the second assessors’ comments and to reach a mutual understanding of the use of categories and when to start or stop coding. Following these discussions, minor adjustments were made to the full sample of observations to ensure consistent use of the categories.

In the further analysis of risky play, only play with great heights, play with high speed, and rough-and-tumble play (R&T) were analyzed in detail, in addition to the category of total risky play, including all risky play categories. This is because the amount of the other categories was quite low (see findings section).

### 2.5. Coding of Play Spaces and Play Materials

Categories used in previous research [66,67,68] were adapted to the context of this study in the process of developing categories for play spaces and play materials for the present analysis. The categories for play spaces included sandbox, pathways, nature, open area, fixed functional play equipment (swings, climbing towers, slides, etc.), fixed role-play equipment (playhouses, boats, huts, stores, etc.), fixed equipment other (tables, storage, etc.), and indoors (cubbies, huts and semi-heated outdoor rooms). Play spaces were coded continuously, and the categories were mutually exclusive.

The use or presence of play materials was coded when a child was holding, using or interacting with a material. To capture the idea that children can use several materials at once, the categories for play materials were not mutually exclusive. The categories for materials were sand, water, mud, nature materials, toys, open-ended materials, and wheeled toys.

The variables for spaces and materials were coded as a percentage of time for each observation. One researcher performed the coding, and a second researcher reviewed a random sample of 10% of the video observations to ensure consistent coding and interpretation.

### 2.6. Analysis

The 935 video observations were equally distributed among boys (*n* = 476) and girls (*n* = 459), the first data collection (*n* = 471, 240 boys and 231 girls) and the second data collection (*n* = 464, 236 boys and 228 girls). To examine associations between risky play, play spaces and materials, multilevel regression was conducted [69]. This analysis was used to control for the hierarchical data structure with observations (*n* = 935), nested within children (*n* = 86), nested within institutions (*n* = 8). Random intercept models for continuous outcomes were used in all regression analysis. VPC calculations for risky play indicated a 0% variance at the institutional level and 6% variance at the child level. Similar variances were found for the subcategories, with a 0% variance at the institution level and 3% variance at the child level for playing with high speed. For play in great heights, there was 0% variance at the institution level and a 4% variance at the child level. Lastly, for rough-and-tumble play, there was 0% variance at the institutional level and 4% at the child level. Two-level models were selected for further analysis following the limited variance and low N at the institutional level.

The categories for risky play were used as dependent variables in the models to explore the association between these forms of play and the outdoor physical environment. Stepwise inclusion of variables starting at the lowest level in the model [70] was performed. An empty model was run first (M0), followed by a model including the space variables (M1). Next, the variables describing the use of materials in the observations were added (M2). Variables describing children’s age and gender were added lastly to the model (M3). Akaike’s Information Criterion (AIC), Deviance, and Schwarz’s Bayesian Information Criterion (BIC) are presented in the models to indicate model fit [70]. Analyses were completed using Stata 14.2 statistical software (StataCorp, College Station, TX, USA). Power analysis was not conducted to determine sample size following the use of mixed modeling and that the measures used were developed in the present study, meaning that the variance estimates were unknown before the analysis was conducted.

## 3. Results

Table 1 shows that the mean amount of risky play in the observations was 13.2% (*SD* = 28.1). Risky play was distributed among play in great heights (4.8%, *SD* = 17.9), play with high speed (5.6%, *SD* = 19.1), play with dangerous tools (0.4%, *SD* = 5.5), rough-and-tumble play (1.8%, *SD* = 10.8), play with impact (0.3%, *SD* = 3.5) and vicarious play (0.3%, *SD* = 3.2). The categories for play where children can get lost and play near dangerous elements were not observed and therefore not coded in this sample.

### 3.1. Risky Play

The full model for risky play (M3) indicates that there is a positive association between risky play and use of nature, fixed equipment for functional play, other fixed structures, and wheeled toys (Table 2). The amount of risky play in the observation is estimated to be 14% higher when children are in nature the entire observation (100%). Using fixed equipment for functional play the full observation is associated with an increase in risky play by 38%, and other fixed structures by 10%. Using wheeled toys, the entire observation is estimated to increase risky play by 6%. Moreover, a positive association between age and risky play is found, and being one year older is associated with an increase in the amount of risky play in the observation by 3%. There is no significant association between gender and risky play. For risky play, M1 (*p* < 0.001), M2 (*p* < 0.01), and M3 (*p* < 0.05) are improved models compared to the previous using a likelihood-ratio test.

### 3.2. Play with High Speed

The final model for play with high speed (M3) indicates a positive association between play with high speed and use of fixed equipment for functional play and wheeled toys (Table 3). The amount of play with high speed in the observation is estimated to be 23% higher when children are on fixed equipment for functional play the entire observation (100%). Using wheeled toys, the full observation is estimated to increase high-speed play by 9%. There is no significant association between play with high speed and children’s gender or age. For high-speed play, M1 (*p* < 0.001) and M2 (*p* < 0.001) are improved models compared to the previous using a likelihood-ratio test. However, M3 is not a significant improvement over M2.

### 3.3. Play with Great Heights

The final model for play with great heights (M3), shown in Table 4, indicates that there is a positive association between play in great heights and fixed functional equipment and other fixed structures. The proportion of play in heights in the observation is predicted to be 15% higher when children are using fixed equipment for functional play the entire observation (100%). Using other fixed structures is estimated to increase the amount of playing in heights by 11%. There is no significant association between playing in heights and age or gender. For play in heights, M1 (*p* < 0.001) and M2 (*p* < 0.001) are significantly improved models compared to the previous model using a likelihood-ratio test. M3 is not a significant improvement compared to M2.

### 3.4. Rough-and-Tumble Play

Table 5 shows that the final model for rough-and-tumble play (M3) indicates that rough-and-tumble play is not positively associated with any spaces or materials in the outdoor environment. Boys are estimated to have 2% more rough-and-tumble play in each observation compared to girls. There is also a positive association between age and rough-and-tumble play, and being one year older is estimated to increase the amount of rough-and-tumble play in the observation by 2%. For rough-and-tumble play, M1 is not a significant improvement, while M2 (*p* < 0.05) and M3 (*p* < 0.001) are improved models compared to the previous using a likelihood-ratio test.

## 4. Discussion

The descriptive results in this study (Table 1) show that children engage in risky play on average 13% of the outdoor free play time in ECEC. As already reported from other parts of this project, the amount of risky play is equal to other common types of play such as symbolic/dramatic play, and almost all children seem to engage in risky play to some level [71]. The present results show that the risky play mostly consisted of play with high speed and play in great heights, and some rough-and-tumble play. These are all play types that include movement and physical activity and could be seen in accordance with earlier studies finding physical active and functional play to be the most common on outdoor playgrounds [51,67].

Looking more into how particular play features in the playground afford different kinds of risky play, the results show a positive association between risky play in total and use of nature, fixed equipment for functional play, other fixed structures, and wheeled toys (Table 2). This means that children predictively engage in risky play on play equipment designed for physical activity such as climbing, sliding, and balancing (e.g., climbing towers and slides) as well as wheeled toys that one would expect afford high speed. Moreover, they also use other fixed structures such as outdoor tables and chairs, sheds, and toy storage buildings, not intended for play, to create risky play. This is similar to Sandseter’s [23] previous finding that children tend to climb anything that can be climbed.

When analyzing which type of risky play was associated with the different features in the play environment, the results (Table 3, Table 4 and Table 5) show that fixed equipment for functional play is positively associated with play with high speed and play in great heights. This indicates that children climb, balance and slide with high speed in play equipment such as climbing towers, slides and swings—equipment intended for such activities. Furthermore, other fixed structures were only associated with play in great heights, meaning these were mostly used for play such as climbing and balancing [23], while wheeled toys were only associated with play with high speed, often including bicycles, trolleys, or large play cars. These findings are in accordance with what Obee et al. [61] indicated in their study, but the present study establishes these associations on a larger data material using multilevel regression analysis. In this study, open areas and pathways were found to be weakly, positively related to playing with high speed (M1 in Table 3). The predicted positive effect of using pathways and open areas for playing with high speed is, however, diminished when children’s use of materials is included in the model (M2 in Table 3). Specifically, this finding indicates that children’s engagement in play with high speed in open areas and on pathways is related to using wheeled toys. This finding highlights the interplay between different environmental features in children’s physically active play, as previously demonstrated in research [46,72]. Although pathways and open areas seem to facilitate some risky play with high speed through children’s use of wheeled toys in the present sample, the predicted effect of these spaces is relatively weak. One explanation for this would be that riding bikes and trolleys on pathways usually does not involve enough speed for it to be coded as risky play. Another explanation could be that ECEC practitioners restrict high speed on pathways and open areas because of the risk of crashing into other children or objects along the path, a known worry among practitioners when children play with high speed [23]. Even if ECEC practitioners are not included in this study, previous research suggest that they play an important role in how children are allowed to use the environment while playing [73]. Thus, an implication of this study’s findings suggest that ECEC practitioners should reflect on how to best facilitate children’s exploration and opportunities for challenging play in their institutions, rather than simply controlling and restricting their play.

In this study, being in nature was found to be positively associated with the total amount of risky play in the observation (Table 2). The natural environments in the present study usually included areas with trees, bushes, tree stumps, muddy, grassy hills, and uneven surfaces. Children often prefer to play in these unstandardized spaces [52,53], and they afford higher risk-taking in play than ordinary standardized playground equipment [56]. The findings in this study support the notion that natural environments facilitate risky play. However, when looking at each type of risky play, none of them was significantly associated with nature. This finding may be attributed to the relatively limited amount of observed time in the natural environment (Table 1), following the varying access to nature across the participating institutions.

The results show that rough-and-tumble play is not significantly associated with any of the play spaces or materials, except for a negative association with wheeled toys. The strongest positive association between rough-and-tumble play and space is with nature, even though it is not statistically significant. This is probably an indication that soft surfaces such as grass and soil afford this kind of play, in line with findings that soft surfaces also afford rough-and-tumble play in indoor environments [74]. That no specific spaces in the outdoor environment were significantly related to rough-and-tumble play may indicate that this form of play may evolve in a variety of outdoor spaces. Moreover, rough-and-tumble is seen as a predominantly social type of play [75], and it might be that play involving chasing, fencing, and play fighting is less dependent on specific equipment. Therefore, the possibilities of the physical environment do not seem to be decisive for rough-and-tumble play to occur. Rather, other children and social acceptance of such play among the staff [76] could be more essential. Regardless, a soft surface is probably favorable for play fighting [74], and several of the participating centers lacked soft surfaces outdoors. Additionally, the observations were done in the fall, and due to cold and wet weather, children were dressed in ways that reduced their mobility, which might make rough-and-tumble less attractive in general.

There are limitations to this study. It draws on cross-sectional data that is based on video observations with a duration of two minutes conducted within the children’s everyday environment in a Norwegian context where, in many cases, children’s risky play is supported by teachers and practitioners [16,73,77]. Studies in other cultural contexts with a different perception and practice concerning children’s risk-taking and with other outdoor physical environments could reveal other results. Nevertheless, the Norwegian context is suitable for exploring risky play and its natural occurrence due to the emphasis on free play generally and outdoor play particularly. Moreover, the participating institutions’ social context, like the educators’ practices and education, attitudes, and rules towards risky play, influences children’s possibilities to engage in risky play. A limitation to the present study is the lack of control of essential aspects for children’s engagement in risky play like the social context and teacher and child characteristics.

In line with the UN’s General Comment [2] which identified obstacles to fulfill the UN Convention’s requirements [1] on children’s right to play, autonomy and agency, children’s risky play must be acknowledged and facilitated. An overly focus on safety and restrictions of children’s attempts to meet challenges and risks would be detrimental to children’s free play. The results from this study can therefore be valuable in the way they show how the physical environment can support children’s risky play within challenging, yet safe contexts. This study demonstrates that children utilize possibilities for risky play in the ECEC outdoor environment when allowed, and how children’s risky play is associated with features of the physical environment. The findings show that children utilize affordances of both spaces and materials for risky play and that different affordances (e.g., pathways and wheeled toys) facilitate risky play. These findings have implications for policy, ECEC managers, ECEC teachers, architects, landscape architects and other stakeholders in the field. Children’s play environments should be designed and equipped in ways that give access to various spaces and materials that afford challenging play.

This study’s results are probably highly influenced by Norway’s cultural context, where children are allowed to engage in risky play. Future research should investigate similar relationships in other cultural contexts and explore how the social environment influences how children are allowed to utilize opportunities in the physical environment for different types of risky play.

## 5. Conclusions

This study shows that children engaged in risky play in 13% of the observed time of their outdoor free play in ECEC. Furthermore, children engaged in six out of eight previously defined risk categories, indicating varied interests and/or possibilities. Analysis of the environment revealed that risky play was positively associated with fixed equipment for functional play, nature and other fixed structures, while analysis of play equipment showed that risky play was positively associated with wheeled toys. These results indicate that children take advantage of the available environment and equipment and use it for playing with risk. The knowledge about how specific environments and equipment are suited for particular types of risk can support practitioners in developing their outdoor areas to include varied and exciting play opportunities.

## Figures and Tables

**Table 1:** Descriptive statistics (*n* = 935 observations).

Variable	Mean	*SD*	Min	Max
Child age	4.2	0.7	2.9	5.8
Total risky play	13.2%	28.1	0	100
Speed	5.6%	19.1	0	100
Heights	4.8%	17.9	0	100
R&T *	1.8%	10.8	0	100
Impact	0.3%	3.5	0	69
Vicarious	0.3%	3.2	0	52
Spaces				
Pathways	4.3%	14.9	0	100
Nature	5.9%	21.6	0	100
Open area	52.2%	41.9	0	100
Fixed functional	15.0%	32.9	0	100
Fixed other	6.6%	20.5	0	100
Materials				
Natural materials	13.7%	30.7	0	100
Open materials	7.3%	23.0	0	100
Wheeled toys	13.3%	32.1	0	100

* Rough-and-tumble play.

**Table 2:** Regression models for risky play (*n* = 935 observations).

Model	M0	M1	M2	M3
Fixed part	Coeff. (*SD*)	Coeff. (*SD*)	Coeff. (*SD*)	Coeff. (*SD*)
Intercept	13 (1)	3 (2)	4 (3)	−12 (6)
Spaces				
Pathways		0.00 (0.06)	−0.02 (0.06)	−0.02 (0.06)
Nature		0.12 (0.05) **	0.15 (0.05) **	0.14 (0.05) **
Open area		0.06 (0.03) *	0.05 (0.03)	0.05 (0.03)
Fixed functional		0.38 (0.03) ***	0.38 (0.03) ***	0.38 (0.03) ***
Fixed other		0.10 (0.05) *	0.11 (0.05) *	0.10 (0.05) *
Materials				
Natural materials			−0.09 (0.03) **	−0.08 (0.03) **
Open materials			0.05 (0.04)	0.04 (0.04)
Wheeled toys			0.05 (0.03)	0.06 (0.03) *
Child variables				
Age				3.4 (1.4) *
Boy				2.7 (2.1)
Random part				
Level 1 Variance	742 (36)	623 (30)	611 (30)	607 (30)
Level 2 Variance	44 (17)	45 (16)	48 (16)	45 (16)
Deviance	8876	8719	8705	8696
AIC	8882	8735	8727	8722
BIC	8897	8773	8780	8785

* *p* < 0.05: ** *p* < 0.01: *** *p* < 0.001., AIC = Akaike information criterion, BIC = Bayesian information criterion.

**Table 3:** Regression models for play with high speed (*n* = 935 observations).

Model	M0	M1	M2	M3
Fixed part	Coeff. (*SD*)	Coeff. (*SD*)	Coeff. (*SD*)	Coeff. (*SD*)
Intercept	6 (1)	−1 (2)	0 (2)	−2 (4)
Spaces				
Pathways		0.05 (0.04)	−0.02 (0.04)	−0.02 (0.04)
Nature		0.00 (0.03)	0.00 (0.03)	0.01 (0.03)
Open area		0.05 (0.02) **	0.03 (0.02)	0.03 (0.02)
Fixed functional		0.23 (0.02) ***	0.23 (0.02) ***	0.23 (0.02) ***
Fixed other		−0.01 (0.03)	−0.01 (0.03)	−0.01 (0.03)
Materials				
Natural materials			−0.04 (0.02)	−0.03 (0.02)
Open materials			−0.05 (0.03) *	−0.05 (0.03) *
Wheeled toys			0.09 (0.02) ***	0.09 (0.02) ***
Child variables				
Age				0.1 (1.0)
Boy				2.2 (1.5)
Random part				
Level 1 Variance	352 (17)	305 (15)	292 (14)	292 (14)
Level 2 Variance	18 (7)	16 (7)	19 (8)	18 (7)
Deviance	8163	8040	8008	8006
AIC	8169	8056	8030	8032
BIC	8184	8094	8083	8095

* *p* < 0.05: ** *p* < 0.01: *** *p* < 0.001., AIC = Akaike information criterion, BIC = Bayesian information criterion.

**Table 4:** Regression models for play in heights (*n* = 935 observations).

Model	M0	M1	M2	M3
Fixed part	Coeff. (*SD*)	Coeff. (*SD*)	Coeff. (*SD*)	Coeff. (*SD*)
Intercept	5 (1)	2 (2)	2 (2)	−3 (4)
Spaces				
Pathways		−0.04 (0.04)	−0.02 (0.04)	−0.02 (0.04)
Nature		0.02 (0.03)	0.02 (0.03)	0.02 (0.03)
Open area		−0.01 (0.02)	−0.01 (0.02)	−0.01 (0.02)
Fixed functional		0.14 (0.02) ***	0.15 (0.02) ***	0.15 (0.02) ***
Fixed other		0.11 (0.03) **	0.11 (0.03) **	0.11 (0.03) ***
Materials				
Natural materials			−0.01 (0.02)	−0.01 (0.02)
Open materials			0.11 (0.02)	0.11 (0.02)
Wheeled toys			−0.02 (0.02)	−0.02 (0.02)
Child variables				
Age				1.1 (0.9)
Boy				−0.5 (1.3)
Random part				
Level 1 Variance	307 (15)	282 (14)	276 (13)	275 (13)
Level 2 Variance	12 (6)	12 (6)	10 (6)	10 (5)
Deviance	8039	7960	7937	7935
AIC	8045	7976	7959	7961
BIC	8059	8014	8012	8024

** *p* < 0.01: *** *p* < 0.001., AIC = Akaike information criterion, BIC = Bayesian information criterion.

**Table 5:** Regression models for rough-and-tumble play (*n* = 935 observations).

Model	M0	M1	M2	M3
Fixed part	Coeff. (*SD*)	Coeff. (*SD*)	Coeff. (*SD*)	Coeff. (*SD*)
Intercept	2 (0)	1 (1)	2 (1)	−6 (3)
Spaces				
Pathways		0.00 (0.02)	0.02 (0.03)	0.02 (0.03)
Nature		0.04 (0.02)	0.04 (0.02)	0.04 (0.02)
Open area		0.01 (0.01)	0.01 (0.01)	0.01 (0.01)
Fixed functional		−0.01 (0.01)	−0.01 (0.01)	−0.01 (0.01)
Fixed other		0.01 (0.02)	0.01 (0.02)	0.01 (0.02)
Materials				
Natural materials			−0.02 (0.01)	−0.01 (0.01)
Open materials			−0.03 (0.02)	−0.03 (0.02)
Wheeled toys			−0.03 (0.01) *	−0.03 (0.01) *
Child variables				
Age				1.5 (0.5) **
Boy				1.9 (0.8) *
Random part				
Level 1 Variance	111 (5)	110 (5)	109 (5)	108 (5)
Level 2 Variance	5 (2)	5 (2)	5 (2)	4 (2)
Deviance	7092	7086	7077	7063
AIC	7098	7102	7099	7089
BIC	7112	7141	7152	7152

* *p* < 0.05: ** *p* < 0.01, AIC = Akaike information criterion, BIC = Bayesian information criterion.

## Data Availability

The video material/data in this study is not available for others than the researchers in this project due to risk of identification of research participants and the Data Protection Official for Research in Norway’s rules for protection of privacy. The Data Protection Official for Research in Norway has also decided that the data has to be deleted within June 2022.
